# A phylogeny of *Disakisperma* and resurrection of *Cypholepis* (Poaceae, Chloridoideae, Cynodonteae, Eleusininae)

**DOI:** 10.3897/phytokeys.279.203849

**Published:** 2026-08-26

**Authors:** Paul M. Peterson, Konstantin Romaschenko, Neil Snow, Yolanda Herrera Arrieta

**Affiliations:** 1 Department of Botany MRC-166, National Museum of Natural History, Smithsonian Institution, Washington, DC 20013-7012, USA Department of Botany MRC-166, National Museum of Natural History, Smithsonian Institution Washington, DC United States of America; 2 Department of Biology, Pittsburg State University, Pittsburg, KS 66762, USA Department of Biology, Pittsburg State University Pittsburg United States of America; 3 Instituto Politécnico Nacional, CIIDIR Unidad-Durango-COFAA, Durango, C.P. 34220, Mexico Instituto Politécnico Nacional, CIIDIR Unidad-Durango-COFAA Durango Mexico

**Keywords:** Classification, *
Cypholepis
*, *
Disakisperma
*, ITS, phylogeny, plastid DNA sequences, Poaceae, systematics, taxonomy

## Abstract

The classification of *Disakisperma* is reevaluated with the study of molecular DNA sequences and morphological characters. We present a phylogeny of four species of *Disakisperma* utilizing eight plastid (*rps3*, *rps16-trnK*, *rps16* intron, *rpoC2*, *rpl32-trnL* spacer, *ndhF*, *ndhA* intron, *ccsA*) and the nuclear ribosomal internal transcribed spacer (ITS) regions investigating 31 species of subtribe Eleusininae, including an increase in the number of DNA markers and sequence data for an additional individual of *Disakisperma dubium*. We present a revised classification, resurrect *Cypholepis* with a new generic description for three species, make two new combinations (*C. eleusine* and *C. obtusiflora*), and recognize *Disakisperma* as monotypic with an amended generic description.

## Abstract (es)

La clasificación de *Disakisperma* se reevaluó con el estudio de secuencias moleculares de ADN y caracteres morfológicos. Presentamos una filogenia de cuatro especies *Disakisperma* utlizando ocho plástidos (*rps3*, espaciador *rps16-trnK*, intrón *rps16*, *rpoC2*, espaciador *rpl32-trnL*, *ndhF*, intrón *ndhA*, *ccsA*) y las regiones espaciadoras transcritas internas ribosómicas nucleares (ITS) que investigan 31 especies de la subtribu Eleusininae y incluyendo un aumento en el uso del número de marcadores de ADN y la secuencia para un individuo adicional de *Disakisperma dubium*. Presentamous una clasificación revisada, resucitamous *Cypholepis* con una nueva descripción genérica para tres especies, hacemos dos nuevas combinaciones (*C. eleusine* y *C. obtusiflora*) y reconocemos a *Disakisperma* como monotípico con descripción genérica enmendada.

## Introduction

As treated most recently, the grass genus *Disakisperma* Steud. of tribe Cynodonteae Dumort. and subtribe Eleusininae Dumort., included four species with paniculate inflorescences composed of several unilateral racemes attached alternately to subdigitally along the rachis, spikelets with multiple florets (4–14), mostly 3-veined lemmas and 1-veined glumes, and dorsally flattened, mostly elliptical caryopses that are shallow to broadly concave on the hilar surface with a pericarp weakly adnate to the endosperm (Snow et al. 2013; Soreng et al. 2022; POWO 2026). The four species share lemmas with three veins and sometimes have remnants of two additional veins near the base. The lemmatal macrohairs of *Disakisperma dubium* (Kunth) P.M.Peterson & N.Snow are acute or rounded apically, whereas the lemmatal macrohairs of *D. eleusine* (Nees) P.M.Peterson & N.Snow, *D. obtusiflorum* (Hochst.) P.M.Peterson & N.Snow, and *D. yemenicum* (Schweinf.) P.M.Peterson & N.Snow are clavicorniculate (apically club-shaped with a pointed tip) [Snow 1996]. The native range of *Disakisperma* was said to include the southwestern USA, Mexico to Central, and South America (*D. dubium*); and Ethiopia to South Africa, and the Arabian Peninsula (*D. eleusine*, *D. obtusiflorum*, and *D. yemenicum*) [POWO 2026], but based on this study (see below) is now restricted to the New World.

*Disakisperma*, based on *Disakisperma mexicana* Steud. (Steudel 1854), was placed in synonymy of *Leptochloa dubia* (Kunth) Nees by Chase and Niles (1962). The biology and taxonomy of *L. dubia* [≡ *Disakisperma dubium*] was studied in some detail by Valls (1978), and the four species currently recognized within *Disakisperma* were previously recognized in different genera: *D. dubium* was first described as *Chloris dubia* by Kunth (1816); *D. eleusine* was first described as *Diplachne eleusine* by Nees von Esenbeck (1841); *D. obtusiflorum* was first described as *Leptochloa obtusiflora* by Hochstetter (1855); and *Eragrostis yemenicum* was described initially by Schweinfurth (1894), placed in the monotypic *Cypholepis* by Chiovenda (1908), who later transferred it to *Eleusine* Gaertn. (Chiovenda 1912), while Phillips (1982) transferred it to *Coelachyrum* Hochst. & Nees and Snow et al. (2013) transferred it to *Disakisperma* as *D. yemenicum*. Thorough descriptions, illustrations and a key to the species previously in *Disakisperma* are in Snow et al. (2013).

Molecular studies determined that *Leptochloa* P.Beauv. *s.l*. (Snow 1997) was polyphyletic, consisting of at least five clades or genera, *Dinebra* Jacq., *Diplachne* P.Beauv., *Disakisperma*, *Leptochloa*, and *Trigonochloa* P.M.Peterson & N.Snow (Peterson et al. 2010, 2012, 2015a, 2016; Aliscioni et al. 2012; Snow and Peterson 2012, 2023; Snow et al. 2013, 2018). *Leptochloa monticola* Chase, *L. malayana* (C.E.Hubb.) Jansen ex Veldkamp and *L. tectoneticola* (Backer) Jansen ex Veldkamp, for which molecular sequences are unavailable, remain incertae sedis. *Leptochloa monticola* likely should be transferred elsewhere based on leaf anatomical studies (Snow, unpubl.). *Dinebra*, *Diplachne*, *Disakisperma*, and *Leptochloa* are members of subtribe Eleusininae whereas *Trigonochloa* is a member of subtribe Perotidinae P.M.Peterson, Romasch. & Y.Herrera (Peterson et al. 2012; Snow and Peterson 2012; Soreng et al. 2022). Previous phylogenies of the four species of *Disakisperma* included an analysis of four plastid (*rpL32-trnL*, *ndhA* intron, *rps16* intron, and *rps16-trnK*) and ITS markers rendering a topology of *D. dubium* (*D. yemenicum* (*D. eleusine* + *D. obtusiflorum*)) [Snow et al. 2013; Peterson et al. 2015a].

Here we present a new phylogenetic analysis of *Disakisperma* using ITS and eight plastid markers (*rps3*, *rps16-trnK*, *rps16* intron, *rpoC2*, *rpl32-trnL* spacer, *ndhF*, *ndhA* intron, and *ccsA*) with the inclusion of additional samples and new sequences, to interpret evolutionary relationships and present a revised classification.

## Material and methods

### Taxon sampling

We sampled 50 individuals, representing 34 total species and 17 individuals of *Disakisperma* representing four species (4/4 = 100%). A complete list of taxa including authorities, voucher information, and GenBank numbers is presented in Appendix 1.

We designed our study to characterize relationships among species of *Disakisperma* and most of the Eleusininae by including species in the following genera: *Afrotrichloris* Chiov., *Astrebla* F.Muell., *Austrochloris* Lazarides, *Chloris* Sw., *Chrysochloa* Swallen, *Coelachyrum*, *Cynodon* Rich., *Daknopholis* Clayton, *Dinebra*, *Diplachne*, *Eleusine*, *Enteropogon* Nees, *Eustachys* Desv., *Leptochloa*, *Lepturus* R.Br., *Schoenefeldiella* P.M.Peterson, *Stapfochloa* H.Scholz, and *Tetrapogon* Desf. Additionally, we included three outgroup species: *Brachychloa schiemanniana* (Schweick.) S.M.Phillips, *Dactyloctenium aegyptium* (L.) Willd., and *Neobouteloua lophostachya* (Griseb.) Gould, all in the Dactylocteniinae P.M.Peterson, Romasch. & Y. Herrera (Peterson et al. 2015a, 2016).

### Phylogenetic methods

All procedures related to the sequencing of the plastid and ITS regions were performed in the Laboratory of Analytical Biology at the Smithsonian Institution. Detailed methods for DNA extraction, amplification, and sequencing are given in Romaschenko et al. (2012) and Peterson et al. (2010, 2012, 2014, 2015a, 2016). Geneious Prime v.2020.1.4 (Kearse et al. 2012) was utilized for contig assembly of bidirectional sequences for all regions; and Muscle (Edgar 2004) to align consensus sequences and adjust the final alignment.

The Bayesian tree was constructed with IQ-Tree 3.0.1 (Nguyen et al. 2015). Support was assessed using the approximate Bayes test (aBayes; Anisimova et al. 2011) and 10,000 bootstrap replicates implemented in program IQ-Tree 3.0.1 (BS; Felsenstein 1985; Nguyen et al. 2015). Support values with aBayes ≥ 0.95 and BS ≥ 95% were interpreted as strong support.

A taxon duplication approach (Pirie et al. 2008; Gillespie et al. 2010; Pelser et al. 2010; Soreng et al. 2010; Peterson et al. 2015a, 2015b, 2016, 2020, 2021a, 2025a, 2025b) was applied to a single individual of *Disakisperma obtusiflorum* for which incongruence of plastid and ITS data was detected. A single accession of *D. obtusiflorum* was assigned two entries in the matrices: one containing only ITS and one containing only plastid sequences. This technique allowed us to identify the placements of the incongruent ITS and plastid sequences in the context of the phylogeny, and to hypothesize multiple origins and elucidate complex evolutionary histories.

## Results

### Phylogenetic analyses

Seventy sequences are newly reported in GenBank and the remaining were previously published sequences (Appendix 1) generated in our earlier studies (Peterson et al. 2010, 2015a, 2016; Snow and Peterson 2012, 2023; Snow et al. 2013, 2018). Our data set is missing 17.5 percent (78/450) of the ITS and plastid sequences (Appendix 1). Sequencing success for each of the nine DNA marker regions is given in Table 1.

**Table 1.** Sequencing success of the nine regions: *rps3*, *rps16-trnK*, *rps16 intron*, *rpoC2*, *rpl32-trnL*, *ndhF*, *ndhA* intron, *ccsA*, and ITS.

**Taxa**	***rps3***	***rps16-trnK***	***rps16 intron***	***rpoC2***	***rpl32-trnL***	***ndhF***	***ndhA intron***	***ccsA***	**Combined plastid data**	**ITS**	**Overall**
Total aligned characters	612	928	940	799	986	765	1167	954	7151	759	7910
Number of sequences (ratio)	37 (74%)	48 (96%)	44 (88%)	24 (48%)	50 (100%)	38 (76%)	43 (86%)	35 (70%)	319 (71%)	50 (100%)	369 (82%)
Number of new sequences (ratio)	14 (28%)	9 (18%)	8 (16%)	0 (0%)	2 (4%)	15 (30%)	10 (20%)	10 (20%)	68 (15%)	1 (2%)	69 (17.25%)

### Phylogeny

**Figure 1.**
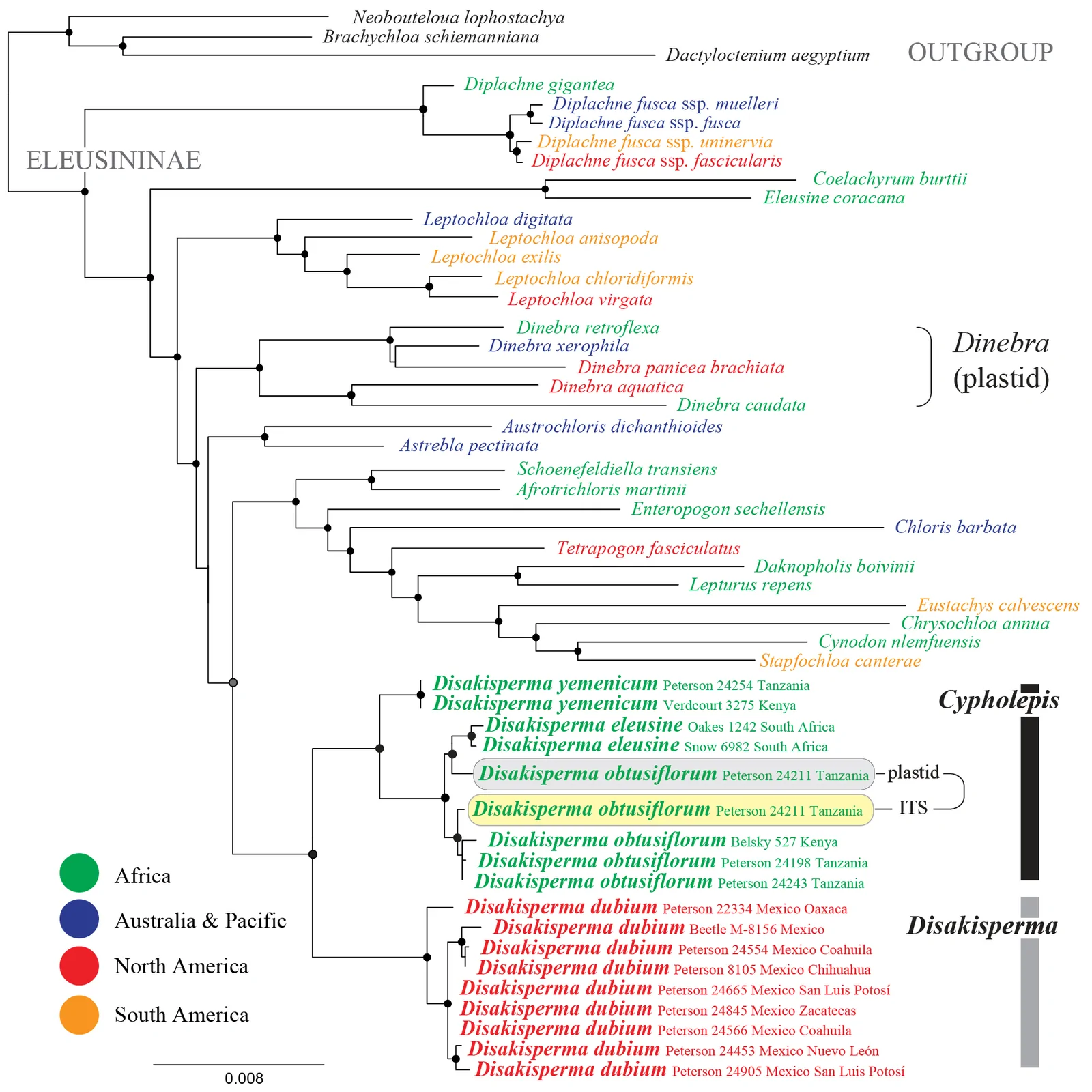
A phylogeny of *Disakisperma* based on plastid (*rps3*, *rps16-trnK*, *rps16* intron, *rpoC2*, *rpl32-trnL*, *ndhF*, *ndhA* intron, *ccsA*) and ITS DNA sequence markers supporting two major clades (*Cypholepis* and *Disakisperma*; and reticulate origins of a single individual of *D. obtusiflorum* from the Tanga Region of Tanzania. Geographic distribution (color); black circles in the phylogram indicate a bootstrap of 95–100 and/or an aBayes of 0.95–1.00. Scale bar: 0.8% substitutions per site.

In our Bayesian tree based on plastid (*rps3*, *rps16-trnK*, *rps16* intron, *rpoC2*, *rpl32-trnL* spacer, *ndhF*, *ndhA* intron, and *ccsA*) and ITS regions we included representatives of most genera in the Eleusininae, and a slightly larger number of species in genera that were formerly placed in *Leptochloa* s.l., excluding *Trigonochloa* since it resides in the Perotidinae (Fig. 1). The first split is the *Diplachne* clade followed by the *Coelachyrum* + *Eleusine* clade, the *Dinebra* clade, and the *Astrebla* + *Austrochloris* clade. Finally, a large clade [*Afrotrichloris* + *Schoenefeldiella* (*Enteropogon* (*Chloris* (*Tetrapogon* (*Daknopholis* + *Lepturus*) (*Eustachys* (*Chrysochloa* (*Cynodon* + *Stapfochloa*))))))))] is sister to the four species of *Disakisperma*.

*Disakisperma* is resolved in two major strongly supported clades (BS = 95–100; aBayes = 0.95–1.00), which are sister groups. One clade is composed of three species (*D. eleusine*, *D. obtusiflorum*, and *D. yemenicum*) and the other is composed of nine individuals of *D. dubium* [Fig. 1]. In the latter clade, *D. eleusine* and *D. obtusiflorum* form a clade that is sister to *D. yemenicum*. One individual of *Disakisperma obtusiflorum* from the Tanga Region of Tanzania (*Peterson, Soreng, Romaschenko & Mbago 24211*) has an ITS sequence that aligns with three other accessions of *D. obtusiflorum* from Kenya and Tanzania in a strongly supported clade, whereas the plastid markers align this same individual with two accessions of *D. eleusine* from South Africa in a strongly supported clade (Fig. 1).

### Taxonomy

#### Cypholepis

Taxon classificationPlantaePoalesPoaceae

(Schweinf.) Chiov., Annuario Reale Ist. Bot. Roma 8(3): 357–358. 1908.

B7FD8E4E-02B9-52E7-82E8-FA341515060D

##### Type.

*Cypholepis yemenica* (Schweinf.) Chiov. ≡ *Eragrostis yemenica* Schweinf. ≡ *Eleusine yemensis* (Schweinf.) Chiov. ≡ *Coelachyrum yemenicum* (Schweinf.) S.M.Phillips ≡ *Disakisperma yemenicum* (Schweinf.) P.M.Peterson & N.Snow.

##### Diagnosis.

*Cypholepis* differs from *Disakisperma* in having only apical panicles that are usually exserted at maturity (v. with apical panicles exserted at maturity and cleistogamous lateral panicles hidden in the lower sheaths) and lemmas with clavate or clavicorniculate hairs (v. lemmas with acute- or round-tipped hairs).

##### Description.

Caespitose ***perennials***. ***Culms*** 30–200 cm tall, 1.0–5.0 mm wide at base, round, erect to sprawling or geniculate, from fibrous or knotted root crowns sometimes bearing short cataphylls (shortly rhizomatous in the opinion of some authors); ***nodes*** glabrous. ***Leaf sheaths*** half as long to slightly longer than internodes, round or slightly flattened, glabrous to occasionally sparsely hairy near apex; ***ligules*** 0.5–2.2 mm long, membranous, apex truncate, ciliate or fimbriate; ***blades*** (3–) 7–37 cm long, flat or sometimes involute or folded upon drying. ***Panicles*** 3.5–65 cm long, narrow 1–3 cm wide (*C. eleusine*, *C. yemenica*) to more open 3–8 (–12) cm wide (*C. obtusiflora*), apical, mostly exserted at maturity, composed of several to numerous unilateral racemes, racemosely or sub-digitately scattered along a central axis; ***branches*** at maturity slightly reflexed to ascending or steeply erect. ***Spikelets*** 3.7–10.0 mm long, 5–12 (–15)-flowered, sessile to subsessile, dorsally rounded to flattened, typically overlapping, disarticulation above the glumes; ***callus*** glabrous; ***glumes*** 2; ***lower glumes*** 1.5–4.1 mm long, 1-veined, membranous; ***upper glumes*** 1.8–5.3 mm long, 1-veined, membranous; ***lemmas*** 2.2–4.7 mm long, ovate, usually 3-veined, sometimes 4- or 5-veined near base (*C. eleusine*, *C. obtusiflora*), membranous, sometimes cartilaginous and with involute margins towards the base (*C. yemenica*), pilose to sericeous below with clavate to clavicorniculate hairs, apex obtuse, sometimes bifid to acute, awnless; ***paleas*** 1/2–2/3 as long as the lemma, short pubescent below, these hairs often clavicorniculate. ***Flowers*** perfect; ***lodicules*** 2, flabellate; ***stamens*** 3; ***anthers*** 0.2–1 mm long, yellow. ***Caryopses*** 1.2–2 mm long, dorsally flattened, broadly concave on the hilar surface; ***pericarp*** weakly adnate to endosperm.

##### Chromosome number.

*n* = 20 (Ammal 1955 for *C. obtusiflora*).

##### Species included.

*Cypholepis eleusine* (Nees) P.M.Peterson & N.Snow, *C. obtusiflora* (Hochst.) P.M.Peterson & N.Snow, and *C. yemenica* (Schweinf.) Chiov.

##### Distribution.

Africa and the Middle East in central Asia (India to Saudi Arabia, Oman, and Yemen).

#### Cypholepis eleusine

Taxon classificationPlantaePoalesPoaceae

(Nees) P.M.Peterson & N.Snow comb. nov.

07C7BF23-241D-5F2F-A1D6-CFF52EB805DC

urn:lsid:ipni.org:names:77395717-1

Diplachne eleusine Nees, Fl. Afr. Austr. 255. 1841 ≡ Uralepis eleusine (Nees) Steud., Syn. Pl. Glumac. 1: 248. 1854 ≡ Tridodia eleusine (Nees) T.Durand & Schinz, Consp. Fl. Afr. 5: 877. 1894 ≡ Leptochloa eleusine (Nees) T.A.Cope & N.Snow, Novon 8: 78. 1998 ≡ Disakisperma eleusine (Nees) P.M.Peterson & N.Snow, Annals Bot. 109: 1327. 2012. Type: South Africa, Katrivierspoort, *J.F.Drège 3906* (lectotype, designated by Snow in Novon 8: 78. 1998: B!; isolectotype: P!).

#### Cypholepis obtusiflora

Taxon classificationPlantaePoalesPoaceae

(Hochst.) P.M.Peterson & N.Snow comb. nov.

F7141637-3B15-53C9-A41A-08B86C07C338

urn:lsid:ipni.org:names:77395718-1

Leptochloa obtusiflora Hochst., Flora 38: 203. 1855 ≡ Eleusine obtusiflora (Hochst.) Blatt., Rec. Bot. Surv. India 8: 505. 1936 ≡ Disakisperma obtusiflorum (Hochst.) P.M.Peterson & N.Snow, Annals Bot. 109: 1327. 2012. Type: Ethiopia, *W. Schimper in Herb. Buchinger 1204* [holotype: STR; isotypes: B!, P!, S! co-mounted with a specimen of Trigonochloa uniflora (Hochst. ex A.Rich) P.M.Peterson & N.Snow].

#### Cypholepis yemenica

Taxon classificationPlantaePoalesGramineae

(Schweinf.) Chiov.

69FE656C-362F-5DDF-8353-6F45C9F656ED

Cypholepis yemenica (Schweinf.) Chiov., Annuario Reale Ist. Bot. Roma 8(3): 357–358. 1908 ≡ Eragrostis yemenica Schweinf., Bull. Herb. Boissier 2 (App. 2): 41. 1894 ≡ Eleusine yemensis (Schweinf.) Chiov., Ann. Bot. (Rome) 10: 410. 1912 ≡ Coelachyrum yemenicum (Schweinf.) S.M.Phillips, Kew Bull. 37(1): 159. 1982 ≡ Disakisperma yemenicum (Schweinf.) P.M.Peterson & N.Snow, PhytoKeys 26: 63. 2013. Type: Yemen, Arabia Felici, 2 Feb 1889, *G.A. Schweinfurth 1332* (lectotype, designated by Snow et al., PhytoKeys 26: 63. 2013 and S.A. Chaudhary, Grasses of Saudi Arabia 274. 1989, cited no specific herbarium: G00164877 [image!]).
= Leptochloa appletonii Stapf, Bull. Misc. Inform. Kew 6: 223. 1907. Type: Somalia, Golis Range, *Drake-Brockman 147* (lectotype designated by Snow et al., PhytoKeys 26: 63. 2013: G [image!]; isolectotype: K000365352 [image!]).
= Eragrostis diplostachya Peter, Repert. Spec. Nov. Regni Veg. Beih. 40 (1, Anhang): 100, t. 58, f. 1. 1929. Type: Tanzania, Lushoto District, Buiko, 14 Jun 1915, *Peter 11083* (lectotype, designated by S.M. Phillips, 59: B. Cypholepis, Gramineae, part 2, Fl. Trop. E. Africa 250. 1974: B10 0168318 [image!]; isolectotype: K-photo!).

### Key to the species of *Cypholepis*

**Table:** 

1	Lemmas cartilaginous below, margins involute near base; adaxial leaf blade with scattered, delicate, straight hairs near the base, the hairs 3–5 mm long; anthers 0.2–0.3 mm long	***C. yemenica***
–	Lemmas membranous throughout, margins not involute near base; adaxial leaf blades surfaces with or without long hairs, the hairs, if present, less than 1.5 mm long; anthers 0.7–1.0 mm long	**2**
2	Lemmas 2.2–2.8 (–3.0) mm long; panicles 3–8 (–12) cm wide; glumes 1.5–2.9 mm long; anthers ca 0.7 mm long	***C. obtusiflora***
–	Lemmas 3.2–4.2 mm long; panicles 1–3 cm wide; glumes 2.8–5.3 mm long; anthers 0.9–1.0 mm long	***C. eleusine***

#### Disakisperma

Taxon classificationPlantaePoalesPoaceae

Steud., Syn. Pl. Glumac. 1: 287 (1854).

27879164-C4AE-5A7E-B623-E861351062F0

##### Type.

*Disakisperma mexicana* Steud. = *Disakisperma dubium* (Kunth) P.M.Peterson & N.Snow ≡ *Chloris dubia* Kunth.

##### Diagnosis.

*Disakisperma* differs morphologically from *Cypholepis* most notably in having cleistogamous inflorescences in the lower leaf sheaths (v. no cleistogamous spikelets in the lower sheaths) and lemmas with acute- or round-tipped hairs (v. lemmas with clavate or clavicorniclate-tipped hairs).

##### Description.

(amended from Peterson et al. 2012 and Snow et al. 2013) Caespitose ***perennials***. ***Culms*** (10–) 30–110 cm tall, 1.0–4.5 mm wide at base, round or flattened, mostly erect or infrequently sprawling, from fibrous root crowns, with hidden (fully inserted) cleistogamous inflorescences in lower leaf sheaths; ***nodes*** glabrous. ***Leaf sheaths*** longer (typically proximally) or shorter (distally) than internodes, typically sparsely hairy below and sometimes densely so near collar, hair bases sometimes tuberculate; ***ligules*** 1.0–1.5 mm long, membranous, truncate and apically short-ciliate; ***blades*** (2–) 8–35 mm long, scabrous near base or sparsely hairy above, glabrous to minutely scabrous below, flat or drying involute. ***Cleistogamous panicles*** completely inserted in lower sheaths. ***Terminal panicles*** 10–45 cm long, (2–)5–25 cm wide, mostly exserted at maturity; ***branches*** composed of (2–) 5–15 unilateral racemes, racemosely or less frequently sub-digitately arising from the rachis, these ascending to reflexed and often slightly flexuous. ***Spikelets*** (4–) 12 mm long, 4–13-flowered, sessile to subsessile, dorsally rounded at maturity, overlapping to distant, disarticulation above the glumes; ***callus*** glabrous; ***glumes*** 2; ***lower glumes*** (1.6–) 2.3–4.8 mm long, 1-veined, membranous; ***upper glumes*** 3.3–5.0 mm long, 1-veined, membranous; ***lemmas*** 4–5 mm long, ovate to obovate or widely obovate, usually 3-veined or sometimes 4–5-veined near base, membranous, sometimes cartilaginous near base, sericeous along lower portions of lateral veins and sometimes on midvein and between veins, hairs acute or rounded apically, apex broadly acute to obtuse or truncate, awnless or mucronate, often bifid; ***paleas*** subequal to lemma, ciliate on margins and sometimes sericeous between veins. ***Flowers*** perfect; ***lodicule*** number not confirmed; ***stamens*** 3; ***anthers*** 1.0–1.6 mm long, yellow. ***Caryopses*** 1.9–2.3 mm long, dorsally flattened, broadly to shallowly concave on the hilar surface; ***pericarp*** weakly adnate to endosperm.

##### Chromosome numbers.

*n* = 20 (Gould 1960; Valls 1978); 2*n* = 40 (Covas 1949; Gould 1960, 1966, 1968), 2*n* = 60 (Brown 1950), 2*n* = 80 (Gould 1960, 1965; Valls 1978).

##### Species included.

*Disakisperma dubium* (Kunth) P.M.Peterson & N.Snow [for complete nomenclatural summary see Snow et al. 2013].

##### Distribution.

Native: In the USA from Arizona to Oklahoma and Texas, and disjunct in southern Florida, in much of Mexico, sporadically in the Caribbean and in Mesoamerica to Bolivia and Chile east to Paraguay, Uruguay, and Argentina; in a variety of vegetation and soil types (including limestone), but most frequently on well drained slopes, mostly 100–2500 m, to 3150 m in the Andes of South America. Non-native: USA in California, Hawaii (Snow and Davidse 2011), Kansas, and Missouri. Its occurrences in the high Andes of Colombia, Ecuador, Bolivia, and Peru may be as introductions (Snow et al. 2013).

## Discussion

*Cypholepis* is supported by our molecular DNA phylogeny, in which all individuals of three species (*C. eleusine*, *C. obtusiflora*, and *C. yemenicum*) are sister to nine individuals of *Disakisperma dubium*. Members of the *Cypholepis* clade have clavicorniculate (apically club-shaped with a pointed tip) hairs on the lemma and contain a single, apical panicle that is exserted at maturity, whereas members of the *Disakisperma* clade, with a single species (*D. dubium*), have acute- or round-tipped hairs on the lemma and contain two panicle types, terminal ones that are completely or mostly exserted and lateral, cleistogamous panicles hidden in the lower sheaths (Snow 1996; Snow et al. 2013). We chose to resurrect the genus *Cypholepis* because of unique morphological synapomorphies characterizing *Cypholepis* and *Disakisperma*, coupled with strong support for genetic separation, and the geographic disjunction of *Cypholepis* in Africa and *Disakisperma* in the New World. However, an alternative would be to recognize the three species of *Cypholepis* as a subgenus within *Disakisperma*.

One individual of *Cypholepis obtusiflora* from the Tanga Region of Tanzania (*Peterson, Soreng, Romaschenko & Mbago 24211*) in our ITS-based phylogeny is embedded in a strongly supported clade with three other individuals of *C. obtusiflora* from Kenya and Tanzania, whereas this same individual, based on its plastid signal, aligns with two individuals of *C. eleusine* from South Africa. Morphologically, the specimen of *Peterson et al. 24211* resembles other members of *C. obtusiflora* given its shorter lemmas [2.2–2.8 (–3.0) mm long versus 3.2–4.2 mm long in *C. eleusine*], wider panicles [3–8 (–12) cm wide versus 1–3 cm], shorter glumes (1.5–2.9 mm long versus 2.8–5.3 mm long), and shorter anthers (ca. 7 mm long versus 0.9–1.0 mm long) [Snow et al. 2013]. We hypothesize that the plastid lineage of the specimen from the Tanga Region was donated from an ancient hybridization event with an individual of *C. eleusine* when these species were sympatric, followed by subsequent introgression involving many individuals of *C. obtusiflora*. Incongruence between nuclear and plastid DNA sequence markers is common in grasses and is prevalent among chloridoid grasses (Peterson et al. 2015a, 2015b, 2016, 2021a, 2025a, 2025b, 2026; Barrett et al. 2020; GPWG III 2024).

The Eleusininae (crown age of 25.85 Ma), a diverse assemblage now consisting of 29 genera with recent additions of *Cypholepis*, *Schoenefeldiella*, and *Pommereulla* L.f., has an ancestral crown area in the Afrotropics (estimated at 90%) and shares a common ancestor with subtribe Dactylocteniinae, also of African origins (Peterson et al. 2015a, 2016, 2021b; Gallaher et al. 2022; GPWG III 2024). *Disakisperma*, treated here as monotypic and restricted to the western hemisphere, appears to be derived from African ancestors since it is sister to *Cypholepis* where all three species are distributed in Africa or central Asia, and *Disakisperma* + *Cypholepis* is sister to a large clade that includes *Afrotrichloris* + *Schoenefeldiella* and *Enteropogon* as the first two splits, where the majority of species are distributed in Africa or central Asia (POWO 2026). The mean crown age of *Cypholepis* and *Disakisperma* appears to be ± 17 Ma (Gallaher et al. 2022).

## Supplementary Material

XML Treatment for Cypholepis

XML Treatment for Cypholepis eleusine

XML Treatment for Cypholepis obtusiflora

XML Treatment for Cypholepis yemenica

XML Treatment for Disakisperma

## Appendix 1

**Table A1.** Taxon voucher (collector, number, and where the specimen is housed), country of origin, and GenBank accession for DNA sequences of *rps3*, *rps16-trnK*, *rps16* intron, *rpoC2*, *rpl32-trnL*, *ndhF*, *ndhA* intron, *ccsA*, and ITS regions; bold indicates new accession; a dash (–) indicates missing data.

	**Taxa**	**Vouchers**	**Country**	***rps3***	***rps16-trnK***	***rps16 intron***	***rpoC2***	***rpl32-trnL***	***ndhF***	***ndhA intron***	***ccsA***	**ITS**
1	*Afrotrichloris martinii* Chiov.	Herlocker 459 (MO)	Somalia	–	**PZ393301**	**PZ377644**	–	**PZ393299**	MK530805	**PZ377604**	MK530789	KP873213
2	*Astrebla pectinata* (Lindl.) F.Muell. ex Benth.	Chalmers 5 (US)	Australia	GU360095	GU360567	GU360311	KX582743	GU359861	GU359585	GU359421	JQ345050	GU359286
3	*Austrochloris dichanthioides* (Everist) Lazarides	Anson s.n. (US)	Australia, Queensland	GU360113	GU360566	GU360310	KX582744	GU359860	GU359584	GU359420	JQ345051	GU359272
4	*Brachychloa schiemanniana* (Schweick.) S.M.Phillips	Schweickerdt 1911 (US)	Mozambique	GU360117	GU360582	–	KX582749	GU359881	GU359776	–	JQ345060	GU359256
5	*Chloris barbata* Sw.	Saarela 1830, Peterson, Soreng & Judziewicz (CAN)	Australia, Northern Territory	MK530874	KP873659	KP873977	KX582751	KP873443	KX582508	KP873838	KX582256	KP873228
6	*Chrysochloa annua* C.E.Hubb.	Arkrok 20417 (US)	Ghana, Yapei Ferri	–	KP873718	KP874002	KX582752	KP873515	KX582509	KP873880	KX582257	KP873300
7	*Coelachyrum burttii* (C.E.Hubb.) P.M.Peterson	Peterson 24163, Soreng, Romaschenko & Abeid (US)	Tanzania, Dodoma	MK530873	KP873646	KP873963	KX582741	KP873429	KX582504	KP873828	–	KP873214
8	*Cynodon nlemfuensis* Vanderyst	Peterson 24058, Soreng, Romaschenko & Abeid (US)	Tanzania, Mbeya	MK530875	KP873742	KP874024	KX582760	KP873542	KX582513	KP873900	KX582262	KP873324
9	*Dactyloctenium aegyptium* (L.) Willd.	Peterson 22283 & Saarela (US)	Mexico, Oaxaca	GU360122	GU360587	GU360432	KX582762	GU359886	GU359713	GU359351	JQ345070	GU359251
10	*Daknopholis boivinii* (A. Camus) Clayton	Ranaivojaona 1441, Andrianjafy, Phillipson & Lubke (MO)	Madagascar, Toliara	MK530876	KP873750	KP874032	KX582765	KP873548	KX582514	KP873907	KX582263	KP873332
11	*Dinebra aquatica* (Scribn. & Merr.) P.M.Peterson & N.Snow	Soderstrom 650 (US)	Mexico, Jalisco	–	KP873751	KP874033	–	KP873549	–	KP873908	–	KP873333
12	*Dinebra caudata* (K. Schum.) P.M.Peterson & N.Snow	Kuchar 24265 (MO)	Tanzania, Singida District	MK530877	JQ345239	JQ345282	MK530834	JQ345324	MK530806	JQ345210	JQ345096	JQ345170
13	*Dinebra panicea* subsp. *brachiata* (Steud.) P.M.Peterson & N.Snow	Peterson 22185 & Saarela (US)	Mexico, Sinaloa	GU360130	–	GU360389	MK530845	GU359810	–	GU359431	–	GU359146
14	*Dinebra retroflexa* (Vahl) Panz.	Snow 6950 & Burgoyne (US)	Eswatini	**PZ377630**	**PZ393302**	–	–	KP873552	**PZ377615**	**PZ377605**	MK530792	KP873336
15	*Dinebra xerophila* (P.M.Peterson & Judz.) P.M.Peterson & N.Snow	Perlman 18438 & Wood (MO)	French Polynesia, Marquesas Isl.	MK530896	JQ345266	JQ345308	MK530853	JQ345353	MK530818	JQ345227	JQ345124	JQ345196
16	*Diplachne fusca* subsp. *muelleri* (Benth.) P.M.Peterson & N.Snow	Badman 1282 (MO)	Australia, South Australia	MK530897	JQ345249	JQ345292	MK530854	JQ345334	MK530819	JQ345216	JQ345106	JQ345181
17	*Diplachne fusca* subsp. *uninervia* (J. Presl) P.M.Peterson & N.Snow	Peterson 20786, Soreng, Romaschenko & Gonzalez-Elizondo (US)	Peru, Arequipa	MK530898	JQ345250	JQ345293	MK530855	JQ345335	MK530820	JQ345217	JQ345107	JQ345182
18	*Diplachne fusca* subsp. *fascicularis* (Lam.) P.M.Peterson & N.Snow	Snow 5996 (KSP)	USA, Missouri	**PZ377631**	MF353873	MF353866	–	MF353857	**PZ377616**	MF353847	**PZ377594**	MF353837
19	*Diplachne fusca* subsp. fusca (L.) P.Beauv. ex Roem. & Schult.	Snow 7249 (KSP)	Australia, Queensland	–	MF353874	MF353867	–	MF353859	–	MF353848	–	MF353839
20	*Diplachne gigantea* Launert	Browning 8 (P)	Botswana, Okavango	–	–	–	–	MF353861	–	–	–	MF353841
21	*Disakisperma dubium* (Kunth) P.M.Peterson & N.Snow	Beetle M-8156 (US)	Mexico	**PZ377632**	**PZ393303**	**PZ393300**	–	**PZ393300**	**PZ377617**	**PZ377606**	**PZ377595**	**PZ371535**
22	*Disakisperma dubium* (Kunth) P.M.Peterson & N.Snow	Peterson 22334 & Saarela (US)	Mexico, Oaxaca	GU360051	GU360695	GU360416	KX582773	GU359811	GU359738	GU359442	JQ345103	GU359145
23	*Disakisperma dubium* (Kunth) P.M.Peterson & N.Snow	Peterson 24453, Romaschenko & Valdés-Reyna (US)	Mexico, Nuevo León	**PZ377633**	**PZ393304**	**PZ377646**	–	KP873555	**PZ377618**	**PZ377607**	–	KP873339
24	*Disakisperma dubium* (Kunth) P.M.Peterson & N.Snow	Peterson 24554 & Romaschenko (US)	Mexico, Coahuila	**PZ377634**	**PZ393305**	**PZ377647**	–	KP873556	**PZ377619**	**PZ377608**	**PZ377596**	KP873340
25	*Disakisperma dubium* (Kunth) P.M.Peterson & N.Snow	Peterson 24566, Romaschenko & Valdés-Reyna (US)	Mexico, Coahuila	**PZ377635**	**PZ393306**	**PZ377648**	–	KP873557	**PZ377620**	**PZ377609**	**PZ377597**	KP873341
26	*Disakisperma dubium* (Kunth) P.M.Peterson & N.Snow	Peterson 24665 & Romaschenko (US)	Mexico, San Luis Potosí	**PZ377636**	**PZ393307**	**PZ377649**	–	KP873558	**PZ377621**	**PZ377610**	**PZ377598**	KP873342
27	*Disakisperma dubium* (Kunth) P.M.Peterson & N.Snow	Peterson 24845 & Romaschenko (US)	Mexico, Zacatecas	**PZ377637**	**PZ393308**	**PZ377650**	–	KP873559	**PZ377622**	**PZ377611**	**PZ377599**	KP873343
28	*Disakisperma dubium* (Kunth) P.M.Peterson & N.Snow	Peterson 24905 & Romaschenko (US)	Mexico, San Luis Potosí	**PZ377638**	**PZ393309**	**PZ377651**	–	KP873560	**PZ377623**	**PZ377612**	**PZ377600**	KP873344
29	*Disakisperma dubium* (Kunth) P.M.Peterson & N.Snow	Peterson 8105 & Annable (US)	Mexico, Chihuahua	**PZ377639**	JQ345247	JQ345290	–	JQ345332	**PZ377624**	JQ345214	JQ345104	JQ345179
30	*Disakisperma eleusine* (Nees) P.M.Peterson & N.Snow ≡ *Cypholepis eleusine* (Nees) P.M.Peterson & N.Snow	Oakes 1242 (US)	South Africa, KwaZulu-Natal	**PZ377640**	KF574422	KF574416	–	KF574410	**PZ377625**	**PZ377613**	**PZ377601**	KF574399
31	*Disakisperma eleusine* (Nees) P.M.Peterson & N.Snow ≡ *Cypholepis eleusine* (Nees) P.M.Peterson & N.Snow	Snow 6982, Burgoyne & Gumbi (MO)	South Africa, KwaZulu-Natal	MK530899	JQ345248	JQ345291	MK530856	JQ345333	MK530821	JQ345215	JQ345105	JQ345180
32	*Disakisperma obtusiflorum* (Hochst.) P.M.Peterson & N.Snow ≡ *Cypholepis obtusiflora* (Hochst.) P.M.Peterson & N.Snow	Belsky 527 (MO)	Kenya	**PZ377641**	JQ345255	JQ345298	–	JQ345340	**PZ377626**	**PZ377614**	JQ345112	JQ345187
33	*Disakisperma obtusiflorum* (Hochst.) P.M.Peterson & N.Snow ≡ *Cypholepis obtusiflora* (Hochst.) P.M.Peterson & N.Snow	Peterson 24198, Soreng, Romaschenko & Mbago (US)	Tanzania, Kilimanjaro	MK530900	KF574423	KF574417	MK530857	KF574411	**PZ377627**	KF574405	MK530793	KF574400
34	*Disakisperma obtusiflorum* (Hochst.) P.M.Peterson & N.Snow ≡ *Cypholepis obtusiflora* (Hochst.) P.M.Peterson & N.Snow	Peterson 24211, Soreng, Romaschenko & Mbago (US)	Tanzania, Tanga	**PZ377642**	KF574424	KF574418	–	KF574412	**PZ377628**	KF574406	**PZ377602**	KF574401
35	*Disakisperma obtusiflorum* (Hochst.) P.M.Peterson & N.Snow ≡ *Cypholepis obtusiflora* (Hochst.) P.M.Peterson & N.Snow	Peterson 24243, Soreng, Romaschenko & Mbago (US)	Tanzania, Tanga	**PZ377643**	KF574425	KF574419	–	KF574413	**PZ377629**	KF574407	**PZ377603**	KF574402
36	*Disakisperma yemenicum* (Schweinf.) P.M.Peterson & N.Snow ≡ *Cypholepis yemenica* (Schweinf.) P.M.Peterson & N.Snow	Peterson 24254, Soreng, Romaschenko & Mbago (US)	Tanzania, Shinyanga	–	KF574426	KF574420	–	KF574414	–	KF574408	–	KF574403
37	*Disakisperma yemenicum* (Schweinf.) P.M.Peterson & N.Snow ≡ *Cypholepis yemenica* (Schweinf.) P.M.Peterson & N.Snow	Verdcourt 3275 (US)	Kenya, Masai	–	KF574427	KF574421	–	KF574415	–	KF574409	–	KF574404
38	*Eleusine coracana* (L.) Gaertn.	Peterson 24112, Soreng, Romaschenko & Abeid (US)	Tanzania, Rukwa	–	KP873758	KP874039	–	KP873566	–	KP873915	–	KP873350
39	*Enteropogon sechellensis* (Baker) T.Durand & Schinz	Peterson 23815, Soreng & Romaschenko (US)	Tanzania, Dar Es Salaam	MK530908	KP873787	KP874064	KX582783	KP873602	KX582531	KP873934	–	KP873386
40	*Eustachys calvescens* (Hack.) Caro & E.A.Sánchez	Chase 10971 (US)	Brazil, Mato Grosso State	–	KP873789	KP874066	–	KP873604	–	KP873936	KX582272	KP873388
41	*Leptochloa anisopoda* (Scribn. ex B.L.Rob.) P.M.Peterson	Steyermark 29192 (US)	Guatemala, Zacapa	–	KP873778	–	–	KP873594	–	–	–	KP873378
42	*Leptochloa chloridiformis* Parodi	Myndel-Pedersen 3741 (US)	Argentina, Corrientes	–	KP873801	–	–	KP873615	–	–	–	KP873402
43	*Leptochloa digitata* (R.Br.) Domin	Risler 476 & Kerrigan (MO)	Australia, Northern Territory	MK530911	JQ345246	JQ345289	MK530867	JQ345331	MK530829	JQ345213	JQ345102	JQ345178
44	*Leptochloa exilis* (Renvoize) P.M.Peterson	Anderson 37049, Stieber & Kirkbride (US)	Brazil, Bahia State	–	KP873676	–	–	KP873464	–	–	–	KP873250
45	*Leptochloa virgata* (L.) P.Beauv.	Rimachi 8359 (MO)	Peru, Loreto Loreto	MK530914	JQ345263	JQ345305	MK530872	JQ345350	MK530831	–	JQ345120	JQ345194
46	*Lepturus repens* (G.Forst.) R.Br.	Peterson 23835, Soreng, Romaschenko & Abeid (US)	Tanzania, Lindi	MK530916	KP873804	KP874076	KX582805	KP873618	KX582545	KP873944	KX582282	KP873405
47	*Neobouteloua lophostachya* (Griseb.) Gould	Peterson 11515 & Annable (US)	Argentina, San Juan	GU360262	GU360725	GU360273	KX582819	GU360004	GU359635	GU359396	JQ345134	GU359123
48	*Schoenefeldiellas transiens* (Pilg.) P.M.Peterson	Peterson 24216, Soreng, Romaschenko & Mbago (US)	Tanzania, Tanga	MK530918	KP873815	KP874088	KX582837	KP873632	KX582565	KP873954	–	KP873415
49	*Stapfochloa canterae* (Arechav.) P.M.Peterson	Pedersen 9597 (US)	Paraguay, Misiones	–	KP873661	KP873978	–	KP873444	–	KP873839	–	KP873230
50	*Tetrapogon fasciculatus* (Hitchc. & Chase) P.M.Peterson	Ekman s.n. (US)	Dominican Republic	GU360171	GU360638	GU360317	KX582846	GU359982	GU359608	GU359528	JQ345142	GU359156

## Additional information

### Artificial Intelligence (AI) use

The authors accept full responsibility for the content of the manuscript, including the disclosure of any use of AI.

No AI tools were used in the preparation of this manuscript.

### Author ORCIDs

P.M. Peterson https://orcid.org/0000-0001-9405-5528

K. Romaschenko https://orcid.org/0000-0002-7248-4193

N. Snow https://orcid.org/0000-0001-8824-7259

Y. Herrera Arrieta https://orcid.org/0000-0003-3814-6518

### Data availability

All of the data that support the findings of this study are available in the main text.
